# Air pollution and cardiovascular diseases: mechanisms, evidence, and mitigation strategies

**DOI:** 10.25122/jml-2025-0018

**Published:** 2025-05

**Authors:** Hari Krismanuel

**Affiliations:** 1 Faculty of Medicine, Universitas Trisakti, Jakarta, Indonesia

**Keywords:** air pollution, cardiovascular diseases, endothelial dysfunction, PM2.5, public health interventions

## Abstract

One of the most urgent environmental health issues is air pollution, which has a major effect on cardiovascular health. Among other pollutants, fine particulate matter (PM2.5) has been connected to a number of cardiovascular illnesses (CVDs), including myocardial infarction, stroke, and hypertension. The purpose of this review was to summarize current research on the processes by which air pollution raises the risk of CVD and to investigate mitigation and preventative measures. A review of peer-reviewed articles published between 2015 and 2025 was conducted using databases such as PubMed, Web of Science, and Google Scholar. The review focused on studies examining the relationship between PM2.5 and cardiovascular diseases, incorporating epidemiological, experimental, and clinical perspectives. PM2.5 and other pollutants exacerbate CVD risk through mechanisms such as autonomic instability, endothelial dysfunction, oxidative stress, and inflammation. Risks are disproportionately high for vulnerable groups, such as the elderly and those with underlying cardiovascular diseases. Targeted public health policies, stricter air quality regulations, and increased public awareness are essential to mitigate the cardiovascular risks posed by air pollution. Immediate action is imperative to safeguard global health.

## INTRODUCTION

Air pollution has become a pressing global health concern, acknowledged as a key factor in non-communicable diseases, particularly cardiovascular diseases (CVDs), which remain the leading cause of mortality worldwide. A substantial body of evidence has established a link between exposure to air pollutants, including fine particulate matter (PM_2.5_), ozone (O_3_), nitrogen dioxide (NO_2_), sulfur dioxide (SO_2_), and carbon monoxide (CO), and the onset and exacerbation of CVD. These substances originate from multiple sources, such as natural processes, vehicle emissions, and industrial activities [1-4]. Significant progress has been made in understanding the detrimental effects of air pollution on cardiovascular health [2,5,6]. However, important research gaps persist, particularly concerning the intricate molecular mechanisms underlying these effects, the long-term consequences for sensitive populations (e.g., children, the elderly, and individuals with pre-existing conditions), and the effectiveness of existing mitigation strategies in diverse environmental contexts [7,8].

Moreover, populations exposed to air pollution exhibit significant heterogeneity, with substantial differences in geographical and socioeconomic factors influencing exposure levels and cardiovascular disease risk. For instance, individuals in developing countries often experience higher exposure to pollution due to biomass combustion and industrial emissions. In contrast, in developed nations, traffic-related pollution and fossil fuel combustion are the predominant sources. Similarly, urban populations tend to face higher air pollution levels than rural populations; however, variables such as healthcare access and socioeconomic status may moderate the impact of pollution on cardiovascular health. These differences can also influence molecular responses to pollutants, with studies showing variations in inflammatory gene expression and oxidative stress responses between urban and rural populations [5,9].

Socioeconomic status (SES) plays a crucial role in determining both exposure levels and health-related outcomes. Existing literature indicates that individuals with low SES are more likely to reside in areas with higher pollution levels and have an increased risk of CVD, potentially due to limited access to healthcare resources and greater exposure to environmental hazards [10].

While urban areas generally experience higher pollution levels, rural regions are not exempt from significant exposure, especially in developing countries. For instance, in rural India and Nepal, biomass combustion for heating and cooking is a major source of household air pollution, contributing to elevated PM_2.5_ levels. Indoor air quality in India exhibits significant spatial variation, with regions differing in climatic conditions, population density, and education levels leading to distinct air quality characteristics. Notably, North Indian states record substantially higher PM_2.5_ concentrations (ranging from 557–601 μg/m^3^) compared to the Southern States (183–214 μg/m^3^). This discrepancy is most likely attributable to the colder climate in Northern Indian areas, where extended periods of low temperatures result in higher heating requirements compared to the warmer climate of southern India. Understanding these population differences is crucial for developing context-specific and efficient mitigation measures. Therefore, effective mitigation strategies must consider these differences and be tailored to the specific contexts of different populations, including factors such as the availability of healthcare services, education, and housing standards [5].

Previous reviews have provided valuable insights into the relationship between air pollution and CVD. However, a comprehensive and up-to-date synthesis of the latest epidemiological, biological, and interventional evidence is lacking. This review seeks to bridge those knowledge gaps by: (1) providing a comprehensive update on the latest epidemiological evidence, with a focus on associations between specific air pollutants (PM_2.5_, O_3_, NO_2_, SO_2_, CO) and various cardiovascular outcomes (e.g., ischemic heart disease, stroke, heart failure); (2) critically evaluating the current understanding of the molecular processes through which these pollutants harm the cardiovascular system, including oxidative stress, inflammation, endothelial dysfunction, and epigenetic modifications, with a focus on the interplay between these mechanisms; (3) assessing the effectiveness of current mitigation strategies, such as emission control technologies and public health interventions, and proposing novel approaches, including personalized exposure reduction strategies and targeted therapies; and (4) identifying key research priorities for future investigations, emphasizing the need for longitudinal studies, multi-pollutant exposure assessments, and research on the long-term health impacts in vulnerable populations [7,11,12].

Specifically, critical gaps in molecular pathways include (a) influence of epigenetic modifications, such as DNA methylation and histone modifications, in modulating long-term cardiovascular impacts of air pollution exposure; (b) how different pollutants interact synergistically to exacerbate oxidative stress and inflammation, particularly ultrafine particles (UFPs) and gaseous pollutants like NO_2_ and SO_2_; and (c) the identification of novel biomarkers that could help elucidate individual susceptibility to air pollution-induced cardiovascular damage. Exploring these mechanisms is key to improving targeted prevention and treatment efforts [13-18].

This review aimed to provide actionable recommendations for policymakers, healthcare professionals, and the public to address the burden of CVD associated with air pollution. This review synthesizes contemporary knowledge regarding the connection between cardiovascular health and air pollution, highlighting molecular pathways such as endothelial dysfunction, oxidative stress, and inflammation. Additionally, it examines practical ideas and methods to reduce air pollution-related CVD burden and influence public health policy and preventive strategies [2,19,20-22].

## MATERIAL AND METHODS

This literature review was conducted to synthesize findings from peer-reviewed articles published between 2015 and 2025. Articles were identified through systematic searches in PubMed, Web of Science, and Google Scholar. The methodology for this review primarily concentrated on research investigating the link between air pollution and cardiovascular illnesses, incorporating epidemiological, experimental (in vivo and in vitro), and clinical perspectives.

The following search terms were used in combination with Boolean operators (AND, OR): ('PM2.5' OR 'particulate matter 2.5' OR 'PM10' OR 'particulate matter 10' OR 'ozone' OR 'O3' OR 'nitrogen dioxide' OR 'NO2' OR 'sulfur dioxide' OR 'SO2' OR 'carbon monoxide' OR 'CO') AND ('cardiovascular disease' OR 'coronary artery disease' OR 'stroke' OR 'heart failure' OR 'hypertension') AND ('air pollution' OR 'environmental pollution').

### Inclusion criteria

- Original research articles (epidemiological studies, experimental studies, clinical trials) published in English.
- Studies investigating the correlation between cardiovascular outcomes and exposure to air pollutants (PM2.5, PM10, O3, NO2, SO2, and CO).
- Studies published between 2015 and 2024.

### Exclusion criteria

- Review articles, editorials, letters to the editor, and conference abstracts.
- Studies not focused on the relationship between air pollution and cardiovascular diseases.
- Studies published in languages other than English.

To determine the eligibility of the retrieved papers, two impartial reviewers looked at abstracts and titles. After that, full texts of articles that were of interest were obtained and evaluated for inclusion using the predetermined standards. Reviewers' disagreements were settled by consensus and discussion. Key findings and study characteristics (author, year, study design, population, exposure assessment, and outcome measures) were extracted using a standardized form.

## RESULTS

### Epidemiological evidence

Epidemiological studies have consistently demonstrated a substantial association between exposure to air pollution and an increased risk of certain CVDs. By employing a range of methodologies, including time-series analyses, case-control studies, and cohort studies, this research provides a comprehensive illustration of the relationship between cardiovascular health and air pollution. Numerous studies have examined particulate matter, particularly fine particulate matter (PM_2.5_), as a significant air contaminant with detrimental effects on the cardiovascular system [19-22].

### Particulate matter (PM2.5 and PM10)

- **Long-term exposure:** Numerous cohort investigations have identified a significant correlation between a higher risk of ischemic heart disease (IHD) and extended exposure to PM_2.5_, which includes myocardial infarction (MI), stroke, heart failure, and cardiovascular mortality. For example, research has indicated that a 10–20% surge in the likelihood of ischemic heart disease and stroke is linked to each 10 μg/m^3^ elevation in sustained PM_2.5_ levels. These results apply to various populations in different locations. Additionally, research suggests that prolonged exposure to PM_10_ is associated with comparable, albeit typically milder, cardiovascular effects [5,7,12,23-25].
- **Short-term exposure:** Short-term increases in PM2.5 and PM10 concentrations have been connected to greater daily hospital admissions and cardiovascular event-related mortality, according to time-series studies. This is particularly true for susceptible groups such as the elderly and people who already have cardiovascular disease. It is believed that mechanisms such as oxidative stress, inflammation, and modifications in autonomic nervous system function cause these acute effects [14,26,27].

### Gaseous pollutants (O3, NO2, SO2, CO) [22,28,29]

- **Ozone (O_3_):** Ozone exposure has also been connected to negative cardiovascular outcomes in epidemiological research, such as an elevated risk of heart failure and stroke. It is believed that inflammation and oxidative stress are the processes by which ozone affects the cardiovascular system.
- **Nitrogen Dioxide (NO_2_):** A higher risk of hypertension, MI, and stroke has been linked to exposure to NO_2_, a sign of air pollution caused by traffic. According to studies, there may be a synergistic negative impact on cardiovascular health from the combined impacts of PM_2.5_ and NO_2_.
- **Sulfur Dioxide (SO_2_):** Although less extensively studied than PM_2.5_ and NO_2_, SO_2_ exposure has also been linked to adverse cardiovascular effects, particularly in susceptible populations.
- **Carbon Monoxide (CO):** CO, a product of incomplete combustion, can reduce the amount of oxygen that the blood can carry, which can exacerbate cardiovascular conditions, particularly in individuals with IHD.

Various air pollutants contribute to the development of cardiovascular diseases through distinct pathophysiological mechanisms, including oxidative stress, inflammation, and endothelial dysfunction. Table 1 summarizes key air pollutants, their primary sources, and their specific cardiovascular effects.

**Table 1:** Summary of pollutants and their cardiovascular effects

Air pollutant	Main sources	Key cardiovascular effects
**PM_2.5_**	Vehicles, industry, wildfires	Hypertension, arrhythmia, atherosclerosis
**O_3_**	Photochemical reactions in the atmosphere	Oxidative stress, inflammation
**NO_2_**	Vehicle emissions, industry	Hypertension, autonomic dysfunction
**SO_2_**	Coal combustion, industry	Vasoconstriction, stroke risk
**CO**	Vehicles, cigarette smoke	Hypoxia, myocardial dysfunction

### Global burden of CVD attributable to air pollution

The worldwide burden of CVDs, which includes heart failure, arrhythmias, MI, stroke, and hypertension, is greatly increased by both brief and prolonged exposure to ambient air pollution. According to studies, cardiovascular diseases account for more than 60% of air pollution-associated morbidity and mortality, outweighing deaths from risk factors related to metabolism, behavior, and tobacco use [24-28]. Chronic diseases like atherosclerosis, hypertension, and stroke are closely linked to prolonged exposure to PM_2.5_. There is still little data on arrhythmias, atrial fibrillation, and heart failure, but what is known suggests that these conditions are positively correlated. Furthermore, particulate matter and gaseous pollutants, including NO_2_, SO_2_, and O_3_, contribute to the development of CVD through mechanisms such as oxidative stress, inflammation, endothelial dysfunction, and autonomic dysregulation [23,24,25,26]. In conclusion, particulate matter and gaseous pollutants pose serious dangers, making air pollution a significant environmental contributor to cardiovascular health. Addressing air quality could reduce the global burden of CVD, highlighting the critical need for environmental interventions and public health measures.

### Mechanism of action

**Figure 1:**
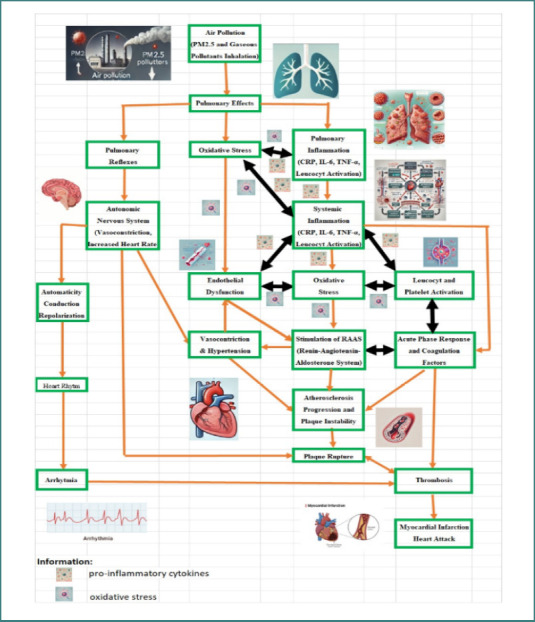
A schematic diagram summarizing the pathophysiological processes connecting cardiovascular diseases to air pollution

There are multiple interrelated pathophysiological pathways through which air pollution contributes to the development and progression of CVDs (Figure 1). Endothelial dysfunction, inflammation, oxidative stress, and disruption of the autonomic nervous system are important pathways. Systemic inflammation can be triggered by gaseous pollutants and PM_2.5_, impairing vascular function, promoting atherosclerosis, and disrupting heart rhythm, all of which contribute to the development and progression of CVD [19,23-28]. There are several intricate pathophysiological pathways that connect air pollution to an increased risk of CVD. Although the exact molecular mechanisms remain incompletely understood, extensive research has identified several significant biological processes linking air pollution exposure to CVD [19,23-28]. These mechanisms include:

- **Inflammation**: Certain gases, such as ozone (O_3_) and air pollutants, especially PM_2.5_ (fine particulate matter), can trigger an inflammatory reaction in the body. Atherosclerosis, characterized by the accumulation of plaque within arteries, stands as a major risk factor for CVD, and the associated inflammation can impact blood vessels and promote its development [19,20-22].
- **Oxidative stress**: Air pollution can cause the body to undergo oxidative stress. An imbalance between the body's capacity to eliminate damaging reactive oxygen species (ROS) and their creation leads to oxidative stress. This oxidative stress can damage the walls of blood vessels, induce inflammation, and accelerate the progression of cardiovascular disease [19,23-28].
- **Endothelial dysfunction**: Exposure to air pollution can cause problems with the inner lining of blood vessels, known as the endothelium. Endothelial dysfunction plays a crucial early role in the development of atherosclerosis, primarily because it contributes to vascular constriction, increased blood pressure, and diminished blood flow [23-28].
- **Autonomic nervous system imbalance**: Air pollution may affect the autonomic nervous system, which regulates heart rate and blood pressure. Changes to this system brought on by pollution may increase the likelihood of arrhythmias (abnormal cardiac rhythms) and other cardiovascular disease-related issues [23-28].
- **Blood coagulation and thrombosis**: Exposure to air pollution is linked to both an increase in blood coagulation and the onset of thrombosis. This could raise the chance of strokes and heart attacks because of thrombosis or the buildup of clots within blood vessels [23-28].
- **Blood pressure elevation**: Air pollution has been linked to both short-term and long-term increases in blood pressure, particularly concerning PM_2.5_. The cardiovascular system may be strained by high blood pressure, potentially raising the likelihood of developing hypertension, which is a significant risk factor for CVD [23-28].
- **Accelerated atherosclerosis**: Atherosclerosis, a condition that narrows arteries and increases the chance of strokes and heart attacks, can have its progression hastened by extended exposure to air pollution [23-28].
- **Impaired cardiac function**: Studies have shown that exposure to air pollution can impair cardiac function by promoting cardiac arrhythmias and altering heart rate variability. Such consequences could elevate the risk of experiencing sudden cardiac events [23-28].
- **Systemic effects**: The respiratory system is not the only part of the body affected by air pollution. Pro-inflammatory cytokines and other compounds that may affect the cardiovascular system are released in this way [23-26].

It is essential to note that the exact mechanisms can vary according to an individual's susceptibility, the type and composition of air pollutants, and the duration and intensity of their exposure [5,23,24]. In addition to other risk factors like smoking and diet, the combined effects of several pollutants can increase the risk of cardiovascular diseases. Addressing the causes of air pollution and lowering exposure to it would help mitigate these harmful cardiovascular effects and continue to be essential public health objectives [5,28,29].

### Effects on cardiovascular diseases

#### Mortality and morbidity from all causes of CVD

Heart-related mortality is associated with prolonged exposure to air pollutants, including ground-level ozone (O_3_) and fine particulate matter (PM_2.5_). This category of fatal outcomes encompasses strokes, heart attacks, heart failure, and other heart-related conditions. Contributing variables include inflammation, oxidative stress, and exacerbation of preexisting cardiovascular conditions [21,22,30-32]. Air pollution raises rates of morbidity as well as the risk of dying from cardiovascular disease. Elevated levels of air pollution are associated with an increased incidence of numerous cardiovascular episodes that do not result in death, such as heart attacks, strokes, angina (chest pain), and hospitalizations for heart-related disorders [21,22,30-32].

#### Myocardial infarction and ischemic heart disease

Inflammation and oxidative stress can be brought on by exposure to air pollutants, particularly O_3_ (ground-level ozone) and PM_2.5_ (fine particulate matter). This can cause arterial plaques to build and break, which can lead to a heart attack [29-34]. Individuals already suffering from cardiovascular diseases, including conditions like coronary artery disease or congestive heart failure, are more susceptible to aggravated symptoms upon exposure to air pollution. By decreasing the oxygen supply and increasing the heart's workload, pollutants can strain the heart [21,29-34].

#### Atherosclerosis and arterial stiffness

The narrowing and hardening of arteries brought on by the buildup of plaque—which is made up of calcium, fat, cholesterol, and inflammatory cells—is known as atherosclerosis. Blood clots may form as a result of this constriction, which lowers blood flow [21]. Long-term exposure to air pollution, particularly fine particulate matter (PM_2.5_), might hasten atherosclerosis. The endothelium, the lining that surrounds blood vessels, can be harmed by pollutants, and they can also encourage the buildup of fatty deposits, which reduces the artery's flexibility and increases its susceptibility to blockages [21].

#### Blood pressure and hypertension

One of the main risk factors for ischemic heart disease is elevated blood pressure, or hypertension, which is linked to exposure to air pollution. Hypertension increases the likelihood of cardiovascular disease by placing additional strain on the heart [21].

#### Heart failure

Air pollution is responsible for the onset and exacerbation of heart failure, a chronic cardiovascular illness in which the heart's ability to pump blood efficiently is impaired [33]. Multiple biological mechanisms link air pollution to the development and progression of heart failure:

1. **Inflammation and oxidative stress**
  Air pollution, particularly ozone (O_3_) and fine particulate matter (PM_2.5_) can cause the body to experience oxidative stress and inflammation. These reactions can lead to systemic inflammation, which affects the heart and blood vessels. Prolonged inflammation and oxidative stress can damage the heart muscle and accelerate the development of heart failure [19,20].
2. **Worsening of pre-existing conditions**
  Individuals with pre-existing cardiovascular diseases, such as hypertension, coronary artery disease, or a history of myocardial infarction, are more vulnerable to the harmful effects of air pollution. Continued exposure can accelerate the progression of these conditions and increase the risk of developing or worsening heart failure [34-38].
3. **Reduced oxygen supply**
  High concentrations of air pollutants, such as PM_2.5_, for example, can reduce the blood's capacity to carry oxygen. The heart must exert greater effort to supply enough oxygen to the tissues of the body, particularly its muscles. The heart's increasing workload can eventually lead to heart failure by causing the heart's chambers to enlarge [38-42].
4. **Elevated blood pressure**
  Hypertension, or elevated blood pressure, is linked to air pollution. Hypertension can cause the heart to work harder, resulting in left ventricular hypertrophy. This may ultimately result in cardiac failure by making it more difficult for the heart to pump blood effectively [43-45].
5. **Impaired endothelial function**
  Air pollution can have an adverse effect on the blood vessel lining's endothelial cells. This endothelial dysfunction may result in lower blood flow to the cardiac muscle and constriction of blood vessels. Sustained impairment of endothelial function may worsen heart failure [46,47].
6. **Arrhythmias**
  The heart's electrical signaling can be disrupted by air pollution, leading to arrhythmias (irregular heartbeats). Heart failure risk can be raised by arrhythmias, which can also reduce the heart's effective blood pumping capacity. Atrial fibrillation (AF), a common arrhythmia, manifests as swift and irregular heartbeats originating in the heart's upper chambers, known as the atria. Air pollution, especially O_3_ and PM_2.5_, can contribute to AF development through processes such as autonomic nervous system dysfunction, oxidative stress, and inflammation [34,48-51]. In addition to the effects on heart rate and rhythm, the elevated risk of arrhythmias such as AF can reduce the heart's capacity to effectively pump blood and raise the risk of heart failure. Because they cause potentially fatal changes in the electrical activity of the heart, such arrhythmias may, at times, trigger cardiac arrest, particularly in people who already have heart issues [34,48-51].

In addition to direct pathophysiological effects, air pollution may indirectly contribute to heart failure by exacerbating pre-existing cardiovascular conditions, including ischemic heart disease and hypertension [2,52,53]. Furthermore, air pollution can also reduce an individual's exercise tolerance, discouraging physical activity that is vital for heart health. Moreover, chronic exposure to polluted air has been linked to reduced life expectancy, with cardiovascular disease—particularly heart failure—being a major contributing factor [54--60].

### Vulnerable populations

Certain groups, such as children, the elderly, and those with underlying cardiovascular conditions, are disproportionately affected negatively by air pollution. When exposed to contaminated air, these individuals are more likely to experience severe cardiovascular consequences [5,61,62]. Mitigating these health risks and improving cardiovascular outcomes can be achieved by minimizing exposure to air pollution through regulatory actions, lifestyle modifications (such as reducing time spent outside on days with high pollution), and switching to cleaner energy sources [5,61,62].

## DISCUSSION

### Interpretation of key findings

Our thorough analysis of epidemiological data consistently reveals a strong and alarming correlation between air pollution exposure and a significantly increased risk of several cardiovascular diseases. The validity of these findings is strengthened by the observation of this link across various study designs, including cohort, case-control, and time-series analyses [21,63]. The obvious dose-response link between chronic exposure to fine particulate matter (PM_2.5_) and the incidence of stroke and IHD is a particularly concerning finding. Research has repeatedly shown that for every 10 μg/m^3^ increase in PM_2.5_ concentrations, the risk of these serious cardiovascular events increases by 10–20%. This mathematical relationship highlights the significant negative effects of long-term exposure to air pollution on cardiovascular health and underscores the urgent need for effective measures to reduce PM_2.5_ levels, particularly in densely populated urban areas where exposure is often highest [24,61].

Additionally, our analysis shows that long-term exposure is not the only way that air pollution can have negative impacts. Short-term increases in PM_2.5_ and PM_10_ concentrations have been linked to higher daily hospital admissions and cardiovascular event-related mortality, according to time-series studies. This acute impact is particularly concerning for vulnerable individuals, such as the elderly and those with pre-existing CVD, emphasizing the need for timely public health advisories and targeted interventions during periods of elevated pollution levels to mitigate these acute risks [25,26].

The global burden of CVD attributable to air pollution is staggering. According to studies, cardiovascular diseases account for more than 60% of air pollution-related morbidity and mortality, outnumbering deaths from other significant risk factors such as metabolic disorders, behavioral variables, and even tobacco use. This concerning figure underscores the significance of air pollution as a major environmental risk factor for cardiovascular disease globally, highlighting the need for immediate and comprehensive measures to mitigate air pollution and protect public health worldwide [63-65].

The negative effects of air pollution on the cardiovascular system are caused by a variety of pathophysiological mechanisms that include intricate interactions between biological processes. According to mounting data, the primary mechanisms linking air pollution to the onset and progression of CVD include inflammation, oxidative stress, endothelial dysfunction, and autonomic dysregulation. Exposure to PM_2.5_ and gaseous pollutants increases the risk of a number of cardiovascular diseases, such as IHD, stroke, heart failure, and arrhythmias, by causing systemic inflammation, vascular dysfunction, atherosclerosis (the accumulation of plaque in arteries), and heart rhythm disruption. Developing focused therapies to prevent and treat cardiovascular disease caused by air pollution requires an understanding of these underlying mechanisms [27,42,43].

Lastly, our review emphasizes how some vulnerable communities are disproportionately affected by air pollution. When exposed to polluted air, children, the elderly, and people with pre-existing cardiovascular diseases are far more likely to suffer from catastrophic cardiovascular consequences. This discrepancy highlights the need for targeted initiatives to protect these vulnerable populations, including promoting indoor air quality measures, issuing public health advisories during high pollution levels, and ensuring equitable access to quality medical care. These actions are necessary to mitigate the disproportionate impact of air pollution on individuals who are most vulnerable to its adverse effects [20,64,65].

### Mitigation strategies

A multifaceted strategy that incorporates both preventative and intervention techniques is needed to lessen the harmful cardiovascular consequences of air pollution. These strategies can be broadly categorized into primary, secondary, and intervention strategies, addressing different stages of exposure and disease development.

**1. Primary prevention: reducing emissions at the source**

Primary prevention is the most effective long-term strategy for mitigating the cardiovascular burden of air pollution. It focuses on minimizing air pollutant emissions at their source, thereby reducing population exposure and preventing the initial harm. Key strategies, while promising, face various implementation challenges [66-68]:

**a.Emission control from transportation**

Stricter car emission regulations, such as the European Union's Euro 6 requirements, have been shown to significantly reduce harmful pollutants, including nitrogen oxides (NO_x_) and particulate matter (PM_2.5_). In particular, compared to Euro 5 standards, the nitrogen oxide (NO_x_) emissions from light diesel vehicles have decreased by 55% as a result of the adoption of Euro 6 standards. Furthermore, these rules have reduced PM_2.5_ emissions by up to 99%, which lowers the risk of asthma, lung cancer, stroke, and ischemic heart disease [69-71]. However, even with these standards, real-world emissions often exceed test values due to factors like driving conditions and vehicle maintenance [72]. Promoting the use of electric and hybrid vehicles, although beneficial, faces challenges such as concerns about battery range, limitations on charging infrastructure, and the environmental impact of battery manufacturing, including the extraction of lithium and other rare earth minerals [73,74]. Investing in public transportation infrastructure, encouraging cycling and walking through the development of dedicated infrastructure can further reduce reliance on private vehicles. However, this requires substantial upfront investment and may face resistance due to existing urban planning priorities or lack of public support. For instance, project cost escalations can undermine public support and hinder policymakers' ability to achieve transportation investment goals [75,76]. Additionally, many public transportation systems continue to face challenges, such as declining funding, labor shortages, and limited public resources, which can impede the successful implementation of such infrastructure projects [76,77]. Furthermore, while active commuting (cycling, walking) offers cardiovascular benefits through increased physical activity, it is crucial to acknowledge that during their commute, people may be exposed to higher levels of air pollution, especially in areas with high traffic. Therefore, urban planning strategies should prioritize creating safe and low-pollution routes for cyclists and pedestrians, possibly through the development of green corridors or traffic calming measures [78,79].

**b.Industrial emission reduction**

Implementing stricter regulations on industrial emissions, promoting the adoption of cleaner technologies, and incentivizing energy efficiency can significantly reduce industrial contributions to air pollution, thereby reducing the population's risk of cardiovascular disease linked to industrial pollutants. For example, cap-and-trade programs for sulfur dioxide (SO_2_) emissions have demonstrated success in reducing acid rain and are likely to have had positive impacts on cardiovascular health, although quantifying these specifically can be complex [80-83]. In the European Union, emission reduction policies have been implemented through stringent regulatory frameworks, such as the Industrial Emissions Directive (IED), which mandates Best Available Techniques (BAT) for pollution control, ensuring a reduction in harmful emissions from industrial sources. While effective, these regulations impose significant compliance costs on industries, which may lead to economic trade-offs, including potential job losses or shifts in industrial operations. Similarly, in China, the relationship between the development of green technologies and the reduction of carbon dioxide emissions has been moderated in large part by environmental restrictions. A study by Chang *et al*. found that investment-based regulatory instruments (IER) policies were the most effective in promoting green knowledge innovation (GKI), leading to sustained reductions in carbon dioxide emissions. In contrast, expenditure-based regulation (EER) had a weaker effect and sometimes encouraged firms to adopt short-term cost-cutting strategies. Additionally, command-and-control regulations (CER) were found to have a moderate impact, reinforcing the need for a balanced regulatory approach. Moreover, the spatial spillover effects of green technological innovation on emissions in neighboring regions further highlight the importance of well-designed regulatory policies [82,84]. These results underscore the need for customized regulatory strategies that foster long-term technical breakthroughs in addition to promoting carbon reductions. However, different regions have varying levels of success with these programs due to differences in economic development and industrial structures, presenting challenges in achieving uniform air quality improvements nationwide [82,84]. However, these regulations can be costly for industries, potentially leading to job losses or relocation, creating a tension between economic and public health concerns. Balancing economic concerns with public health benefits is a key challenge. Furthermore, ensuring compliance with regulations requires robust monitoring and enforcement mechanisms, including regular inspections and penalties for violations [84,85].

**c.Transition to clean energy sources**

For long-term improvements in air quality and cardiovascular health, a shift away from fossil fuels and toward renewable energy sources, such as solar, wind, and geothermal power, is crucial [86,87]. This transition can significantly reduce emissions of greenhouse gases and air pollutants linked to cardiovascular disease. For example, studies have shown a correlation between increased renewable energy use and a decrease in hospitalizations for cardiovascular events [88,89]. However, the transition to clean energy faces hurdles, including high initial investment costs, intermittency issues with renewable sources, and the need for grid infrastructure upgrades [90,91]. Additionally, it is necessary to consider and mitigate the environmental impacts of the lifecycle of renewable energy technologies, such as the mining of rare earth minerals for wind turbines and solar panels [92,93].

**d.Urban planning and green infrastructure**

Implementing urban planning strategies that prioritize green spaces, promote natural ventilation, and mitigate the effects of urban heat islands will help reduce air pollution levels in cities, ultimately benefiting the cardiovascular health of city dwellers. Urban forests and green roofs are examples of green infrastructure that can serve as natural air filters, removing pollutants and enhancing air quality [94,95]. However, implementing such strategies requires careful urban planning, community engagement, and long-term investment, which can be challenging in rapidly developing urban areas. Furthermore, the distribution of green spaces within cities is often uneven, with lower-income neighborhoods having less access, which can potentially exacerbate health disparities and environmental injustice [96,97].

**2. Secondary prevention: minimizing exposure and early detection**

Secondary prevention focuses on minimizing individual exposure to air pollution and promoting early detection of cardiovascular effects. This involves strategies to reduce exposure before significant health damage occurs and to identify early signs of cardiovascular disease related to air pollution. While promising, these strategies face a range of implementation challenges [98-100]:

**a.Educating on the importance of maintaining indoor cleanliness to reduce dust and other particles**

This education aims to raise public awareness about the importance of maintaining indoor cleanliness to reduce exposure to dust, particles, and other pollutants that can be harmful to health. This education can include information on how to clean rooms properly, choosing safe cleaning products, and the importance of good ventilation [101,102].

**b.Air quality monitoring and public communication**

People can be empowered to make well-informed decisions about their activities, especially during times of high pollution, by implementing reliable air quality monitoring systems and providing the public with real-time air quality information. For instance, people can modify their outdoor activities based on real-time information on air pollution levels provided by the Air Quality Index (AQI), which is utilized in the US and other nations [103,104]. Similarly, systems like the 'Plume Labs' app in Europe provide localized air quality information, enabling users to avoid high-pollution areas [105,106]. However, the efficacy of these systems is contingent upon the precision and availability of air quality data. There is little air quality monitoring in many underdeveloped nations, hindering the ability to provide timely and accurate information to the public. Furthermore, even with accurate information, behavior change can be a challenging process. Studies have shown that while some individuals modify their activities during high-pollution days, others do not due to factors such as a lack of awareness, inconvenience, or economic constraints [105,106]. Effective public health campaigns are crucial to overcoming these challenges and should address barriers to behavior change, such as lack of trust in authorities, economic pressures to work outdoors, or cultural norms about outdoor activities, while also tailoring information to specific populations (e.g., language, education level) [107,108]

**c.Personal protective measures**

Promoting the use of appropriate respiratory protection, such as N95 masks, during periods of high pollution can reduce individual exposure. Studies have shown that N95 masks can filter out a significant portion of particulate matter, reducing personal exposure. However, the widespread and consistent use of masks can be challenging due to factors such as cost, discomfort, and limited availability. In many developing countries, N95 masks are expensive and not readily accessible to the general population [109,110]. Furthermore, a proper fit and correct usage are essential for mask effectiveness, as improper use can significantly reduce their protective capacity. Policy limitations, such as the lack of subsidies for masks or public health campaigns promoting their use, can also hinder adoption [109,110].

**d.Early detection and screening**

Implementing targeted screening programs for individuals at high risk of developing cardiovascular diseases due to exposure to air pollution can facilitate early detection and intervention. For example, some cities with high air pollution levels have implemented programs to screen individuals with pre-existing conditions for early signs of cardiovascular damage. However, implementing such programs requires significant resources and infrastructure, as well as the development of specific screening tools and protocols. Economic costs can be a significant barrier, particularly in resource-constrained settings [68,111]. Furthermore, identifying individuals at high risk due to air pollution exposure can be complex, as other factors also contribute to the risk of cardiovascular disease [112]. Developing effective screening tools for air pollution-related cardiovascular changes is challenging, as the effects of air pollution can be subtle and difficult to distinguish from other risk factors [113]. Furthermore, access to healthcare facilities and trained personnel for conducting screenings can be limited, particularly in rural or underserved areas [114].

**e.Indoor air quality improvement**

Improving indoor air quality is a crucial secondary mitigation strategy to reduce exposure to air pollution, particularly for vulnerable populations. This can be achieved through non-technological approaches such as increasing natural ventilation, using exhaust fans in enclosed areas, and optimizing building designs to minimize pollutant accumulation. Additionally, behavioral modifications—such as reducing indoor emission sources (e.g., tobacco smoke and the use of biomass fuel)—can further improve indoor air quality [115-117]. However, these strategies face several challenges, including structural limitations in older buildings that hinder proper ventilation and the absence of stringent building codes requiring adequate air circulation [118,119]. Addressing these barriers may require policy interventions that promote better ventilation standards and public awareness campaigns on maintaining clean indoor air [120,121]. Table 2 compares these mitigation strategies based on their regulatory frameworks, lifestyle interventions, public health initiatives, and overall effectiveness.

**Table 2:** Comparison of primary and secondary mitigation strategies for air pollution

Air pollution mitigation strategies				
Mitigation strategy	Regulatory policies	Lifestyle interventions	Public health initiatives	Effectiveness
**Primary mitigation**				
Regulatory Policies	Emission control regulations (e.g., Clean Air Act)	Promotion of active transport (walking, cycling)	Government subsidies for clean energy adoption	High – Directly reduces pollution at the source but requires enforcement and economic investment
Transition to Clean Energy	Industrial air quality standards	Reducing fossil fuel dependence (e.g., electric vehicles)	Public campaigns to reduce emissions	High – Long-term air quality improvements, reducing cardiovascular events
Urban Planning & Green Infrastructure	Vehicle emission limits	Development of green infrastructure (e.g., urban forests)	Incentives for green infrastructure	High – Significantly reduces pollution by improving urban environments and absorbing pollutants
Air Quality Monitoring & Communication	Urban zoning laws to reduce pollution	Public awareness of pollution exposure risks	Health advisories for vulnerable populations	Moderate – Enables informed decisions, reducing exposure to high pollution levels
**Secondary mitigation**				
Personal Protective Measures	Indoor air quality regulations (e.g., ventilation standards)	Use of air purifiers, wearing masks	Public education on pollution risks	Moderate – Helps reduce personal exposure but does not eliminate pollution source
Early Detection & Screening	Policies on pollution alerts (e.g., air-based warnings)	Wearing masks	Public education on pollution risks	Moderate – Facilitates early cardiovascular disease detection and intervention
Indoor Air Quality Improvement	Regulations on indoor pollution (e.g., tobacco smoke)	Dietary modifications to improve respiratory resilience	Medical screenings and respiratory interventions	Moderate – Reduces cardiovascular risks through improved air quality

**3. Intervention strategies: managing health impacts**

This section focuses on steps taken to mitigate the negative effects of current air pollution on people's health, especially for those who are susceptible or have pre-existing conditions. Instead of stopping pollution at its source, the focus is on managing and adapting to the pollution that already exists. These tactics are designed to mitigate the adverse cardiovascular effects of air pollution on individuals who have been previously exposed to it [110,122,123].

Below is a breakdown of the proposed intervention strategies:

**a. Technological interventions:**

**•Indoor air quality improvement**

Technological solutions, such as air purifiers with HEPA filters, have been scientifically proven to reduce indoor air pollution levels, particularly fine particulate matter and allergens. Studies have shown that HEPA filtration can significantly improve indoor air quality and provide measurable health benefits [124,125]. For example, fourteen cross-over RCTs (18 publications) found that individuals with cardiovascular diseases who used HEPA air purifiers at home experienced a -2.28 mmHg [95% CI, -3.92 to -0.64] reduction in systolic blood pressure, indications of improvements in reactive hyperemia index (RHI) (0.10 [-0.04 to 0.24]), diastolic blood pressure (-0.35 [-1.52 to 0.83] mmHg), pulse pressure (PP) (-0.86 [-2.07 to 0.34] mmHg), and C-reactive protein (-0.23 [-0.63 to 0.18] mg/L) following indoor air purification compared to those who did not use air purifiers [126].

Despite these benefits, the cost and maintenance of HEPA filters remain a significant barrier, particularly for low-income households [127]. Additionally, the following variables determine the effectiveness of air purifiers: proper usage, filter replacement schedules, and room size compatibility [128]. Policy-driven interventions—such as subsidies, financial incentives, or public health programs—could encourage the adoption of air purification technology in vulnerable communities to enhance accessibility [129].

**b. Behavioral interventions:**

**•Health advisories and behavioral recommendations**

Issuing health advisories to the public, particularly to susceptible populations such as children, the elderly, and those with respiratory and cardiovascular disorders, can help reduce outdoor activities during periods of poor air quality. This can minimize exposure and prevent exacerbation of cardiovascular symptoms [79,110,130,131]. For example, early warning systems implemented in Beijing have enabled individuals to reduce their exposure during high pollution episodes, leading to a quantifiable percentage reduction in emergency room visits for asthma and other respiratory conditions that can trigger cardiovascular events [132]. However, public health advisories are only effective if the information is accurate, accessible, and trusted by the public. Language barriers, limited access to technology, and distrust of authorities can hinder the dissemination and uptake of information [133]. Furthermore, behavior change can be challenging due to social norms, lifestyle factors, and economic constraints [134]. In addition, guidance on protecting oneself from exposure to pollutants, such as using appropriate masks (N95 or equivalent) and avoiding areas with heavy traffic, can also be effective [135,136].

**c. Healthcare system interventions:**

This strategy focuses on managing existing health conditions to prevent them from worsening due to exposure to air pollution. Some actionable steps include [110,130,137]:

**•Clinical guidelines development**

Developing specific clinical guidelines for managing respiratory (asthma, COPD) and CVDs (coronary heart disease, stroke) that consider the impact of air pollution. These guidelines should include diagnostic, treatment, and monitoring strategies tailored to the specific conditions of pollution exposure [83]. However, developing and implementing effective clinical guidelines requires collaboration between healthcare professionals, researchers, and policymakers, which can be complex and time-consuming [138].

**•Improved access to healthcare**

Ensuring easy and affordable access to healthcare for vulnerable populations is crucial in managing cardiovascular complications related to air pollution. Studies have shown that improved access to healthcare can lead to better disease management and reduced hospital admissions for cardiovascular conditions [68,139]. However, financial constraints, inadequate healthcare infrastructure, and shortages of medical professionals—especially in underserved communities—often limit access to these critical services [140,141]. Implementing proactive health outreach programs to identify and manage high-risk patients can further reduce the burden of cardiovascular diseases related to air pollution. These programs may include community-based screenings for hypertension and heart disease, telemedicine consultations for at-risk populations, and educational initiatives promoting lifestyle modifications to mitigate the harm caused by air pollution to heart health [68,139].

**•Rehabilitation programs**

Providing pulmonary and cardiac rehabilitation programs for patients with chronic diseases to improve their organ function and quality of life, even in polluted environments. For example, a study in Minnesota, USA, found that cardiac rehabilitation programs tailored to patients with cardiovascular disease and exposed to high levels of air pollution improved their exercise capacity and quality of life, which can have positive long-term effects on cardiovascular health [142,143].

**•Patient education and self-management support**

•Educating patients on how to manage their cardiovascular conditions in polluted environments, including strategies to minimize exposure and optimize medication adherence, is essential. For instance, patient education programs that inform individuals with hypertension or coronary artery disease about the increased risks associated with air pollution can empower them to take proactive steps. This includes understanding how to monitor their blood pressure, recognize symptoms of angina or heart failure exacerbation, and adjust their activity levels based on air quality forecasts [144].

•Supporting patients in adopting heart-healthy lifestyle modifications is critical. This includes guidance on dietary choices that promote cardiovascular health, tailored exercise regimens that consider air quality levels (e.g., exercising indoors on high-pollution days), and comprehensive smoking cessation programs. Smoking significantly exacerbates the negative impact of air pollution on the cardiovascular system, making cessation a vital component of risk reduction. Furthermore, education on stress management techniques is also important, as stress can negatively impact heart health and be compounded by the stress of living in a polluted environment [145,146].

•Providing education on the importance of regular check-ups and early intervention when symptoms arise is also crucial [147].

**d. Personalized medicine approaches:**

Utilizing genetic, epigenetic, and other omics data to identify individuals more susceptible to the effects of pollution and tailor interventions accordingly is a promising area of research. For example, researchers are exploring the use of genetic markers to identify individuals who are at a higher risk of developing cardiovascular disease due to exposure to air pollution, which could lead to more targeted prevention and treatment strategies. However, personalized medicine approaches, while promising, face challenges related to cost, data privacy, and the need for further research to validate the effectiveness of tailored interventions [148].

Developing more targeted and personalized therapies based on individual characteristics [149].

Various intervention strategies have been developed to mitigate the health impacts of air pollution exposure. Table 3 summarizes these approaches, categorized into technological interventions, behavioral interventions, healthcare system interventions, and personalized medicine approaches. Each strategy varies in effectiveness depending on implementation factors, population coverage, and suitability of individual risk profiles. While technological and public health policy interventions tend to provide broad benefits, personalized medicine offers more targeted interventions based on genetic and environmental factors.

**Table 3:** Intervention strategies for reducing health risks from air pollution

Intervention Strategies	Technological Interventions	Behavioral Interventions	Healthcare System Interventions	Personalized Medicine Approaches	Effectiveness
**Regulatory Measures**	- Advanced air filtration systems in industries	- Promotion of active transport (walking, cycling)	- Government enforcement of air quality standards	- Genetic screening for pollution susceptibility	**High** - Directly reduces pollution at the source but requires strong enforcement and investment
**Transition to Clean Energy**	- Development of clean energy technologies (e.g., hydrogen fuel)	- Reducing fossil fuel dependence (e.g., electric vehicles)	- Public campaigns promoting renewable energy	- Targeted health interventions for high-risk genetic profiles	**High** - Long-term air quality improvements, reducing hospitalizations
**Urban Planning & Green Infrastructure**	- Smart city air monitoring sensors	- Community engagement in green infrastructure	- Incentives for urban greening projects	- Personalized recommendations based on pollution exposure risk	**High** - Significantly reduces pollution by improving urban environments
**Air Quality Monitoring & Communication**	- Al-driven air pollution prediction models	- Public awareness campaigns on pollution risks	- Health advisories for vulnerable populations	- Individualized pollution exposure tracking apps	**Moderate** - Enables informed decisions, reducing health risks
**Personal Protective Measures**	- Wearable air purifiers	- Use of face masks in high-exposure areas	- Distribution of masks during high pollution days	- Tailored respiratory protection strategies for susceptible individuals	**Moderate** - Helps reduce personal exposure but does not eliminate pollution sources
**Early Detection & Screening**	- Biomarker-based pollution exposure testing	- Encouraging routine health check-ups	- Public health screening programs	- Genomic-based risk assessment for air pollution-related diseases	**Moderate** - Facilitates early disease detection and intervention
**Indoor Air Quality Improvement**	- Smart home air purification systems	- Dietary modifications for respiratory resilience	- Medical screenings for pollution-related illnesses	- Precision medicine approaches for lung disease prevention	**Moderate** - Reduces exposure to indoor air pollutants, lowering health risks

### Integration of mitigation and intervention strategies

It is crucial to emphasize that intervention strategies do not replace mitigation strategies (i.e., actions taken to reduce pollution at the source). They are complementary and address different aspects of the air pollution problem. Primary mitigation efforts to reduce pollution at the source remain the most effective long-term solution for reducing air pollution and its associated health risks, including cardiovascular disease. These strategies aim to prevent the harmful effects of air pollution before they occur, thereby offering the greatest potential for long-term improvements in population health [23,27,68]. Meanwhile, intervention strategies are essential for protecting public health now and mitigating the adverse effects of existing pollution. Even with aggressive mitigation efforts, it will take time to significantly reduce ambient air pollution levels. Intervention strategies, such as providing access to air purifiers for vulnerable populations, developing clinical guidelines for managing air pollution-exacerbated cardiovascular conditions, and educating individuals on how to reduce their exposure, are crucial for minimizing harm to those currently exposed to unhealthy air. They address the immediate needs of individuals already suffering or at high risk due to existing pollution levels [150].

The integration of mitigation and intervention strategies is, therefore, essential for a comprehensive approach to addressing the cardiovascular burden of air pollution. While mitigation strategies offer a long-term solution by addressing the root cause of the problem, intervention strategies provide crucial short-term relief and protection for vulnerable individuals. A balanced approach that prioritizes both mitigation and intervention is necessary to protect public health both now and in the future. For example, while transitioning to electric vehicles (a mitigation strategy) is crucial for long-term air quality improvement, providing access to air purifiers and personalized medicine approaches (intervention strategies) for vulnerable populations is essential to protect their health in the present [88]. Furthermore, research into personalized medicine approaches can inform mitigation strategies by identifying those most susceptible to harm from specific pollutants, allowing for more targeted and effective emissions reductions. This integrated approach maximizes the impact of resources and provides the most comprehensive protection against the harmful effects of air pollution [148].

### Policy and regulatory frameworks

The effective implementation of these mitigation strategies requires a strong policy and regulatory framework. These include the development of air quality standards and regulations that establish and enforce stringent air quality standards for key pollutants [137,151]. Additionally, cross-sectoral collaboration is crucial for fostering collaboration among government agencies, industry, and the public to implement comprehensive air pollution mitigation strategies [152,153]. Finally, promoting international cooperation to address transboundary air pollution issues is also crucial [154,155]. By implementing a comprehensive and integrated approach that encompasses primary, secondary, and tertiary prevention strategies, we can enhance public health and successfully mitigate the harmful cardiovascular effects of air pollution [156,157].

### Limitations

It is important to acknowledge several limitations of this review. Firstly, while we have strived to include a comprehensive range of studies, the available literature on the specific link between certain air pollutants (e.g., specific ultrafine particles or less commonly studied gaseous pollutants) and certain cardiovascular outcomes (e.g., specific types of arrhythmias or heart failure subtypes) may be limited. Publication bias may result from this, as studies with statistically significant positive results have a higher chance of getting published [9,158]. Secondly, variations in study design, exposure assessment methods, and outcome definitions across the included studies may introduce heterogeneity, making direct comparisons and meta-analyses challenging. For example, some studies relied on ambient air pollution measurements from fixed monitoring stations, which may not accurately reflect individual exposure levels, while others used more sophisticated exposure modeling techniques. Similarly, definitions of cardiovascular outcomes may vary across studies, potentially affecting the comparability of results [159,160]. Thirdly, while epidemiological studies can demonstrate associations between air pollution and cardiovascular diseases, they cannot definitively establish causality. Confounding variables may affect both exposure to air pollution and the risk of cardiovascular illnesses. These include socioeconomic status, lifestyle habits (such as smoking, nutrition, and physical activity), and pre-existing medical disorders. While many studies attempt to control for these confounders, residual confounding cannot be entirely ruled out [161].

### Implications and future research directions

The conclusions of this review have important ramifications for policy and public health. The strong and consistent evidence linking air pollution to cardiovascular diseases underscores the urgent need for comprehensive plans to reduce air pollution and protect public health. These strategies should be prioritized:

- Strengthening air quality regulations by implementing and enforcing stricter air quality standards for key pollutants, particularly PM_2.5_, NO_2_, and O_3_, is crucial [129,134,162].
- Promoting clean transportation and energy by investing in sustainable transportation infrastructure, promoting the adoption of electric vehicles, and transitioning to renewable energy sources are essential for reducing emissions from key sources [163,164].
- Raising public awareness by educating the public about the health risks of air pollution and empowering individuals to take steps to minimize their exposure is vital [165,166].
- Implementing targeted interventions to protect vulnerable populations, such as children, the elderly, and individuals with pre-existing cardiovascular conditions, is necessary to reduce health disparities [167].

Despite significant advancements in understanding the link between air pollution and cardiovascular diseases (CVDs), several research gaps remain. Addressing these gaps is crucial for developing more effective mitigation strategies and interventions. Key areas for future research include:

### Investigating the specific effects of less commonly studied pollutants

- Further research is needed to investigate the specific cardiovascular effects of ultrafine particles (UFPs <0.1 µm), specific gaseous pollutants, and pollutant mixtures [64,168].
- Longitudinal cohort studies assessing the chronic effects of UFP exposure on cardiovascular health [64,168].
- Mechanistic studies to determine how UFPs interact with cellular pathways leading to oxidative stress and systemic inflammation [64,168].
- Exploring the synergistic effects of pollutant mixtures, particularly how UFPs interact with gaseous pollutants such as NO_2_ and SO_2_, to exacerbate endothelial dysfunction, vascular inflammation, and oxidative stress, ultimately increasing cardiovascular disease risk [64,168].

### Improving exposure assessment methods

- Developing more accurate and personalized exposure assessment methods is crucial for reducing exposure misclassification and improving the precision of epidemiological studies [169-171].
- Advancing real-time personal exposure monitoring through wearable sensors, mobile applications, and geospatial modeling to capture better individual variations in air pollution exposure [169-171].
- Integrating multi-pollutant exposure models that account for complex interactions between particulate matter (e.g., PM_2.5_, UFPs) and gaseous pollutants (e.g., NO_2_, O_3_, SO_2_) to improve risk estimation and intervention strategies [170,171].
- The role of personal exposure monitoring (e.g., wearable sensors, real-time air quality alerts) in reducing individual cardiovascular risk [170,171].
- Investigating the effectiveness of personalized lifestyle interventions, such as targeted exercise plans and dietary modifications, to counteract pollution-induced effects [170,171].
- Enhancing the use of biomarkers and omics-based approaches (e.g., metabolomics, epigenomics) to link short- and long-term exposure to pollution and its effects on cardiovascular health [170,171].

### Conducting longitudinal studies with repeated measurements

- Longitudinal studies with repeated measurements of both air pollution exposure and cardiovascular health outcomes can provide stronger evidence for causal relationships [172].
- Studying how diabetes and hypertension modify the cardiovascular effects of air pollution, particularly in urban populations with high exposure [172,173].
- Integrating multi-omics approaches (e.g., metabolomics, epigenomics, transcriptomics) to capture dynamic biological changes associated with long-term air pollution exposure and identify potential biomarkers of susceptibility [174,175].
- Examining the synergistic effects of air pollution and lifestyle-related risk factors such as physical inactivity, poor diet, and chronic stress in exacerbating cardiovascular disease progression [56,176].

### Investigating gene-environment interactions

- Research exploring the interaction between genetic susceptibility and air pollution exposure can help identify individuals at particularly high risk [23,177].
- Specific genetic polymorphisms, such as *GSTP1, NQO1*, and *CYP1A1*, have been linked to differential susceptibility to air pollution-induced cardiovascular effects. Investigating how these polymorphisms influence oxidative stress and inflammatory responses can provide insights into individual variability in disease risk [178,179,180].
- Epigenetic modifications, particularly DNA methylation changes, have been observed in response to air pollution exposure. These alterations may affect the regulation of genes involved in inflammation, oxidative stress, and vascular function, contributing to long-term cardiovascular risk [181].
- Studies integrating genetic susceptibility models to identify high-risk populations who may benefit from specific preventive strategies [181].
- Exploring how genetic predisposition affects the effectiveness of personalized exposure reduction strategies, including targeted air filtration, dietary modifications, and pharmacological interventions [181].

### Evaluating the effectiveness of interventions

- Rigorous evaluation of the effectiveness of various air pollution mitigation and intervention strategies is crucial for informing policy decisions [182].
- Real-world implementation trials are needed to compare the efficacy of pharmacological interventions (e.g., statins, ACE inhibitors, beta-blockers) with lifestyle-based interventions, such as modifying physical activity patterns (e.g., encouraging indoor exercise on high-pollution days) or dietary adjustments to counteract pollution-induced cardiovascular effects [182].
- Technology-based interventions, like HEPA filters, portable air purifiers, and advanced filtration masks, should be systematically evaluated in clinical and community-based studies to assess their real-world effectiveness in reducing pollutant exposure and mitigating cardiovascular risks [116,126,128,183].
- Clinical trials evaluate whether statins, antioxidants (e.g., vitamin C, vitamin E), or anti-inflammatory drugs can reduce cardiovascular damage from air pollution [27,184].
- Examining how ACE inhibitors and beta-blockers can help mitigate pollution-induced hypertension and autonomic dysregulation [27,184].
- Assessing the comparative effectiveness of personalized vs. population-wide exposure reduction strategies, particularly in individuals with genetic susceptibility to air pollution-related cardiovascular disease [185].

### Research in understudied populations

- More research is needed in developing countries and other understudied populations to better understand the global impact of air pollution on cardiovascular health [186].
- Studies should focus on region-specific pollution sources, such as biomass burning, industrial emissions, and traffic-related pollution, which may differ significantly from pollution profiles in high-income countries. Understanding these variations is crucial for developing context-specific mitigation strategies [186].
- Investigating transgenerational effects, particularly how maternal exposure to high levels of air pollution during pregnancy influences the cardiovascular health of offspring. Epigenetic changes, including DNA methylation and histone modifications, may play a crucial role in mediating these long-term effects [181,187-189].
- Investigating disparities in air pollution exposure and associated health risks among vulnerable populations, such as low-income communities and elderly individuals [190,191].

### Advancing policy and public health strategies

- Evaluating the real-world effectiveness of air quality regulations in reducing cardiovascular disease burden [68,83].
- Long-term policy impact assessments are necessary to determine whether air quality improvements result in measurable reductions in cardiovascular morbidity and mortality. This includes evaluating the effectiveness of emission control policies, clean energy transitions, and urban planning initiatives [68,83].
- Exploring technology-driven interventions, such as smart air quality monitoring systems, AI-based pollution forecasting, and high-efficiency filtration systems, to mitigate exposure at both individual and community levels [68,83].
- Assessing the role of urban planning, green infrastructure, and clean energy transitions on public health outcomes [68,83].
- Investigating the effectiveness of community-based interventions, such as public awareness campaigns, behavioral nudges, and citizen engagement in air quality monitoring, to promote sustainable pollution-reducing behaviors [68,83].

## CONCLUSION

This comprehensive analysis of epidemiological and mechanistic research provides compelling evidence that air pollution significantly exacerbates cardiovascular conditions. Exposure to various air pollutants—particularly fine particulate matter (PM_2.5_)—is closely associated with an increased risk of cardiovascular disease (CVD). This relationship is mediated through multiple complex biological mechanisms, including oxidative stress, endothelial dysfunction, systemic inflammation, and dysregulation of the autonomic nervous system. Findings from large-scale cohort studies, supported by mechanistic investigations, consistently demonstrate these associations. Notably, research indicates that for every 10 μg/m^3^ increase in PM_2.5_ concentration, the risk of IHD increases by approximately 10–20%. Targeted interventions are necessary since this elevated risk is especially noticeable in vulnerable groups, such as the elderly and people with underlying cardiovascular diseases. The substantial contribution of air pollution to the global burden of CVD surpasses that of several other well-established risk factors. Addressing this pervasive global public health challenge requires multifaceted and urgent action. We recommend the urgent implementation of stricter air quality regulations, the accelerated transition to clean energy sources, and enhanced public awareness campaigns to minimize exposure and lessen the damaging effects of air pollution on cardiovascular health. Furthermore, advancing longitudinal research on the long-term effects of ultrafine particles and rigorous evaluation of specific intervention strategies is crucial for refining our understanding and informing evidence-based policy interventions that can protect global cardiovascular health.
